# Mental healthcare services in Kenyan counties: a descriptive survey of four counties in Western Kenya

**DOI:** 10.1186/s12913-023-09481-w

**Published:** 2023-05-25

**Authors:** Edith Kamaru Kwobah, Matthew Turissini, Julius Barasa, Mercy Kimaiyo, Lily Okeyo, Joash Araka, Faith Njiriri, Richard Matundura, Florence Jaguga

**Affiliations:** 1 Moi Teaching and Referral Hospital, P.O BOX 3-30100, Eldoret, Kenya; 2 Department of Internal Medicine, Indiana University, Bloomington, USA; 3 Academic Model Providing Access to Healthcare, PO BOX 4606, Eldoret, Kenya

## Abstract

**Background:**

The government of Kenya has made progressive efforts towards improving mental health services in the country. However there is little documentation of mental health services in the counties that would support actualization of the legislative frameworks in the context of a devolved healthcare system. This study sought to document existing mental health services within 4 counties in Western Kenya.

**Methods:**

We conducted a cross sectional descriptive survey of four counties using the World Health Organization, Assessment Instrument for Mental Health Systems (WHO-AIMS). Data was collected in 2021, with 2020 being the year of reference. We collected data from the facilities offering mental healthcare within the counties as well as from County health policy makers and leaders.

**Results:**

Mental healthcare was provided at higher level facilities within the counties, with minimal structures at primary care facilities. No county had a stand-alone policy on mental health services or dedicated budget for mental healthcare. The national referral hospital, within Uasin-Gishu county, had a clear mental health budget for mental health. The national facility in the region had a dedicated inpatient unit while the other three counties admitted patients in general medical wards but had mental health outpatient clinics. The national hospital had a variety of medication for mental health care while the rest of the counties had very few options with antipsychotics being the most available. All the four counties reported submitting data on mental health to Kenya health information system (KHIS). There were no clearly defined mental healthcare structures in the primary care level except for funded projects under the National referral hospital and the referral mechanism was not well defined. There was no established mental health research in the counties except that which was affiliated to the national referral hospital.

**Conclusion:**

In the four counties in Western Kenya, the mental health systems are limited and not well structured, are faced with limited human and financial resources and there is lack of county specific legistrative frameworks to support mental healthcare. We recommend that counties invest in structures to support provision of quality mental healthcare to the people they serve.

## Background

Mental disorders are common in the world and cause a substantial burden for affected individuals and their families [1]. A systematic review and meta-analysis that included 174 surveys across 63 countries in the world reported that 1 in 5 respondents fulfilled a criteria for at least one common mental disorder in the 12-month period preceding the assessment [2]. According to World Health Organization (WHO), mental, neurological and substance use disorders accounted for 10.4% of global Disability Adjusted Life Years, 28.5% of global Years Lost to Disability (YLDs) and contribute about 17.6% of all years lost to disability in Africa and they contribute to significant economic and social hardships that affect the society as a whole [3].

There is a huge treatment gap for mental illness despite new developments in evidence-based treatment for mental illness, with WHO estimating that up to 75% of those affected in many low-income countries do not have access to the treatment they need [4]. In Kenya, a study done in Nandi county showed that while 191 (45%) of the participants had a lifetime diagnosis of at least one of the mental disorders, only 1.7% of the participants had been on management for a mental illness indicating a high unmet need [5]. This unmet need has negative implications on the individual and the society in-terms of the direct and indirect costs associated with mental illness such as loss of productivity and the effect on economic growth [6].

The government of Kenya has progressively made efforts towards improving mental health services in the country. The first effort was the launch of the Kenya Mental Health Policy 2015–2030 and one of the objectives of the policy is to ensure access to health care services at all levels of healthcare [7]. Recently the government launched the mental health action plan 2021–2025 and the action proposes integration of mental health into primary care settings [8]. In the year 2010, Kenya promulgated a new constitution which saw the decentralization of health care with transfer of responsibility for health service delivery from the central government to the 47 semi-autonomous county governments [9]. Unfortunately, there is little documentation of mental health systems in the counties that would support actualization of the legislative and policy frameworks.

The objective of the current study therefore was to document existing mental health systems within 4 counties in Western Kenya (Busia, Bungoma, Uasin Gishu and Trans-Nzoia Counties), in order to inform ongoing efforts to increase access to mental healthcare services.

## Methods

This was a cross sectional descriptive survey involving four counties within Western Kenya region that are part of the catchment area for ongoing population health efforts within AMPATH (Academic Model Providing Access to Healthcare). AMPATH is a partnership of Moi University (MU), Moi Teaching and Referral Hospital (MTRH), and a consortium of North American universities [10]. AMPATH delivers care, provides education, and performs research in collaboration with various county governments in Western Kenya and Ministry of Health, and has been at the frontline of efforts to increase access to mental health care through capacity building, community education, as well as screening and linkage [11].

Data was collected from four counties within the AMPATH Catchment area; Bungoma Busia, Uasin-Gishu and Trans-Nzoia counties. These counties were selected because there are ongoing collaborations with AMPATH mental health program to increase mental healthcare access. Each of these counties are semi-autonomous governments, each managing it’s own healthcare system with the national government providing policy directions [12]. TransNzoia, Bungoma and Busia each has a county referral hospital. Uasin - Gishu county on the other hand hosts Moi Teaching and Referral hospital, a tertially institution under the national government, hence does not have a referral hospital of it’s own. In the end we obtained detailed information for three county referral facilities (Level 4 hospitals) and one national hospital located within Uasin Gishu county, the Moi Teaching and Referral Hospital, a level six hospital.

Source of data: Information was obtained from various personnel in leadership positions including nurse managers, medical superintendents and the county health officials.

Data collection instrument: The data collection guide was derived from the World Health Organization, Assessment Instrument for Mental Health Systems (WHO-AIMS) [13]. We used the brief version of the tool since the mental health systems in Kenya are generally under-developed. The brief WHO-AIMS has a total of 54 questions touching on each of the six different domains. (I) policy and legislative framework; (II) mental health services; (III) mental health in primary care; (IV) human resources; (V) public education and links with other sectors; and (VI) monitoring and research. For purposes of this study, the items in the WHO-AIMS were framed into actual questions for ease of administration. We developed 2 questionnaires, one with questions targeting county health leadership and a second one with questions targeting the purposively selected county referral hospital leadership.

Data was collected in 2021, with 2020 being the year of reference. The face to face or phone call interviews were conducted by two research assistants with training in social behavioral research. The respondents were re-contacted via phone call in case clarifications were needed or there was incomplete data during data entry.

Data were entered into an excel data base and checked for completeness and accuracy. Data were summarized using descriptive statistics. Continuous data were summarized using means, and categorical data using frequencies and percentages.

## Results

We report findings of data collected from county leadership and four selected facilities on all the six domains of the WHO AIMS.

### Policy and legislative framework

#### Mental health policy and legislation

There was a national standalone mental health policy and a mental health legislation (mental health act 1989).

No county had a county specific policy on mental health services. However, they all referred to the mental health act of 1989. There were no copies of the national mental health policy in any of the counties.

#### Budget allocation for mental health

At the time of data collection there was no evidence provided for specific mental health specific budget at the county level and only two county facilities had a dedicated line budget to cater for mental health services. These were one national referral facility in Uasin Gishu county and one county referral facility in Bungoma County.

### Mental health services

#### Inpatient services

There was one purposely built mental health unit in the region for inpatient care at the Moi Teaching and Referral hospital, a facility with an 80 bed capacity. In TransNzoia, Bungoma and Busia, mental health inpatient services were provided within the general wards. In all the four health facilities, patients were admitted involuntarily, and physical restraining and seclusion was used in the various facilities.

#### Outpatient services

Mental health outpatient services were available in all the four counties. In the four sampled facilities, a total of 12, 220 patients were seen at the mental health outpatient clinics in the FY 2020/2021. Of the total number of patients seen, 43.14% were attended to at the national referral hospital in the region while 56.96% were attended to at the three county referral facilities.

#### Availability of psychosocial interventions

Psychosocial interventions were offered in all the selected facilities (Table 1). These included psychotherapy, psychoeducation, counseling, social support, and rehabilitation activities.

**Table 1:** Availability of Psychosocial Interventions in the Mental Health Facilities

	Psychological intervention available
National Facility 1	Psychotherapy, psychoeducation, provision of social support, rehabilitation activities
County Facility 1	Counselling, provision of social support
County Facility 2	Psychotherapy, psychoeducation, provision of social support, rehabilitation activities
County facility 3	Psychotherapy, psychoeducation, provision of social support

#### Forensic mental health services

Forensic mental health assessments were offered in 3 counties; Uasin Gishu county Bungoma and Trans-Nzoia counties. All four facilities provided treatment for mentally ill offenders on out- patient basis and referred for any inpatient service required.

#### Availability of medication

Medications for the management of mental health disorders were scantily available in the county facilities. While the national hospital had several options of medications, including both new and older medications, the county hospitals had fewer options and the supply was erratic. (Table 2).

**Table 2:** The types of drugs available in the 4 selected facilities in 2020

Medication Category	Medication	No of Facilities stocking drugs	Percentage
Anti-psychotics	Aripiprazole	1	25%
	Quetiapine	1	25%
	Haloperidol	3	75%
	Olanzapine	3	75%
	zuclopenthixol	1	25%
	Fluphenazine	4	100%
	Chlorpromazine	3	75%
	Risperidone	1	25%
Antidepressants	Amitriptyline	4	100%
	Carbamazepine	4	100%
	Quetiapine	1	25%
	Mirtazepine	1	25%
	Fluoxetine	3	75%
	Lithium	1	25%
Anxiolytics	Bromazepam	1	25%
	mirtazapine	1	25%
	midazolam	3	75%
Drugs for OCD	Clomipramine	1	25%
Drugs for alcohol and substance use disorder	Vitamin B,	1	25%
	Bupropion	1	25%
	naltrexone	1	25%
	nicotine replacement therapy	1	25%

### Mental health in primary care

From our survey, mental health services were mainly delivered in county referral facilities. There were a few mental health programs in primary care facilities mostly in facilities that were supported by AMPATH programs and the National referral hospital. Table 3 below shows the number of facilities offering mental health care in the year 2020.

**Table 3:** Number of facilities offering a mental health service by 2020

County	No of facilities with an established mental health service
Uasin Gishu	3
Bungoma	4
Trans Nzoia	1
Busia	1

### Human resource for mental health

Two counties and one national referral facility provided data on their human resource for mental health. Of these, the total number of psychiatrists were 7, Psychiatric nurses 25, occupational therapists 15, social workers 13, psychologists 18 and psychiatric clinical officers 9. (Table 4)

**Table 4:** Number of mental health care providers in three counties in 2020

Mental health staff	National facility 1	Bungoma County	Tans nzoia County	Busia County	Uasin Gishu County
Psychiatric nurse	14	6	5	1	2
Psychiatrist	4	1	2	0	0
Clinical officer psychiatrist	3	6	0	-	-
Social worker	5	2	6	-	-
Occupational therapist	7	2	6	-	-
Psychologist/ Counselor	4	2	12	-	-

### Public education and linkage with other sectors

In all the four counties there were scanty education sessions for mental health in form of continuous medical education for health care providers. One county referral facility from Bungoma conducted monthly Continuous Medical Education (CME) sessions for clinical staff in mental health service delivery. The CMEs covered diagnosis of mental disorders and psychosocial education. Busia and Trans Nzoia counties had erratic education sessions that were dependent on partner’s facilitation. The referral national hospital conducted outreaches to institutions of learning and community based groups, an effort that was supported by both the hospital funds and an externally funded care program.

In all the four counties, higher facilities received referrals from lower-level facilities. Three of the facilities from Uasin Gishu, Bungoma and Trans Nzoia received referrals of patients with mental health conditions from complementary/alternative/ traditional health practitioners.

### Monitoring and research

All the four counties reported submitting data on mental health to Kenya health information system (KHIS). This was done monthly and included the numbers of new and revisit patient encounters. Data submitted included caseloads and patient statistics. One county facility from Trans Nzoia County additionally reported to the national referral facility on severe mental health cases, rehabilitation data and total number of patients at the clinic on a quarterly basis. Data on Mental health disorders in Bungoma, Busia and Trans Nzoia County had been lumped into one without referring to diffrent diagnoses, making it hard to differentiate them and report effectively. Mental health Research was only reported at the national referral hospital in Uasin Gishu county.

## Discussion

This study provides a preliminary evidence base of the existing mental health systems within 4 counties in the western Kenya region. From our study there is minimal legal and legislative framework, scattered mental health services, minimal mental healthcare in primary care facilities, limited resources for mental health and minimal mental health research.

We found no county specific Policy and legislative framework although the Kenyan government has in place a mental health policy 2015–2030 [14], as well as a National Mental Health Action plan [15]. This is in keeping with a comparative study that included South Africa, Ghana, Uganda and Zambia that revealed that the implementation of mental health policies is highly deficient in many African countries due to lack of a clear operationalization plan and limited resources [16]. Poor governance has been identified as a barrier to effective integration of mental health care in low- and middle-income countries [17]. Counties in Kenya, being the custodians of healthcare in a devolved setting, [12] will need to put in place policy and legislative framework to promote and protect the mental health and also develop clear a mental health care system design and quality assurance to ensure optimal policy implementation.

There were inpatient and outpatient services provided at the level 4 hospitals within each county but minimal mental health services were being provided at the primary care level. Mental health integration in primary care level has been shown to be feasible in an African setting as demonstrated in Kenya [18], South African rural clinics [19], and Nigeria [20] as a potential pathway for providing affordable and accessible mental health care, yet this has not been operationalized in many settings in Western Kenya. There is need for counties to take up this challenge and invest in mental healthcare in primary care level in order to achieve Universal healthcare. A study done in Ethiopia, India, Nepal, South Africa and Uganda participating in the PRogramme for Improving Mental health carE (PRIME) suggests that in order to achieve this kind of integration, counties would need to leverage existing services such as HIV or non-communicable disorders which are well established at this level, as well as the networks of community healthcare workers.

There were limited human resources for mental health in the four counties. This is in keeping with an auditors general report that had been done in Kenya revealing that there was a gross shortage of human resources in the country [21]. As an example, the entire country had about 120 registered psychiatrists in 2020, and only five were in Western Kenya. The auditor general’s report recommended that the country needed about 1,441 psychiatrist in order to achieve a good psychiatrist- patient level. Given that healthcare is devolved in Kenya, this number will only be achieved if counties invest in training their own mental healthcare workers. This will require the goodwill of the county leadership and financial investment as well as clear training needs assessment [22].

From our survey, there was minimal continuous education on mental health for the public and the healthcare workers. Lack of awareness on the cause and course of mental illness, as well as the existing treatment options is believed to contribute to the high treatment gap for mental illness [23]. A systematic review from Sub-Sahara Africa demonstrated that traditional beliefs about the cause of mental illness contribute significantly to stigmatizing attitudes hence the need to have continuous awareness campaigns in- order to promote health seeking behavior [24]. Counties will need to create structures to educate the public and staff on mental health and mental illness.

In our survey, we found gaps in reporting of mental health data to the national level, and inadequate data collection and monitoring. This is in agreement with a study done in Ethiopia, India, Nepal, Nigeria, South Africa and Uganda that establishes substantial challenges in mental health Information systems across all the 6 countries [25]. Collection of quality mental health data, processing and reporting is key in planning and improving service delivery, [26] and counties will need to put in efforts to ensure adequate data is not only being collected but information obtained is used to improve services and justify budgetary allocation for mental health.

There is minimal mental health research in the counties except at the national referral hospital. This is in agreement with existing literature which has recognized a dearth of evidence from the African continent and has advocated for putting in structures to change the research landscape in the continent [27]. A study on building capacity for mental health research in Kenya indicated that two of the biggest contributors to low research activities in our settings is lack of resources and overworked healthcare providers who have many other roles including leadership and academic duties, leaving very little room to engage in research [28]. The value of research in informing providers on trends and risk factor of disorders, safety and efficacy of treatments, outcomes of interventions and care cannot be overemphasized [29]. Counties will need to budget for research at their level but also participate in research that is coordinated at the national level.

### Limitations of the study

While this study provides a good reference base for existing mental health systems in the four counties in Kenya, some limitations must be considered while interpreting the findings. First, this was a cross sectional descriptive survey conducted in only four counties hence minimizing generalizations. Second, the process of evaluation is likely to have been influenced by reporting bias as respondents may view this as an audit. Third there was minimum documentation available indicating the services provided hence the information on mental health in the counties is scanty. Fourth we did not get detailed information from the lower level facilities like level 3, level 2 to establish what was being provided. In future, a national survey including all the 47 counties may give a better description of mental health services in Kenyan counties.

## Conclusion

We found that in in Western Kenya, there are limited mental health services, limited human resource for mental health and a clear lack of county specific legistrative frameworks to support mental healthcare. There was scanty data on mental health care being collected in the counties making it difficult to make good conclusions. We recommend that counties consider having county mental health systems that would support the provision of quality mental healthcare. There is need to improve mental health information systems in order to provide data that would support planning, justify and investment and improve service delivery.

## Data Availability

The datasets used and/or analyzed during the current study are available from the corresponding author on reasonable request.
